# Improving peptide-protein docking with AlphaFold-Multimer using forced sampling

**DOI:** 10.3389/fbinf.2022.959160

**Published:** 2022-09-26

**Authors:** Isak Johansson-Åkhe, Björn Wallner

**Affiliations:** Division of Bioinformatics, Department of Physics, Chemistry and Biology, Linköping University, Linköping, Sweden

**Keywords:** machine learning, ML, AI, peptide-protein docking, AlphaFold, improved sampling, peptide-protein, interactions

## Abstract

Protein interactions are key in vital biological processes. In many cases, particularly in regulation, this interaction is between a protein and a shorter peptide fragment. Such peptides are often part of larger disordered regions in other proteins. The flexible nature of peptides enables the rapid yet specific regulation of important functions in cells, such as their life cycle. Consequently, knowledge of the molecular details of peptide-protein interactions is crucial for understanding and altering their function, and many specialized computational methods have been developed to study them. The recent release of AlphaFold and AlphaFold-Multimer has led to a leap in accuracy for the computational modeling of proteins. In this study, the ability of AlphaFold to predict which peptides and proteins interact, as well as its accuracy in modeling the resulting interaction complexes, are benchmarked against established methods. We find that AlphaFold-Multimer predicts the structure of peptide-protein complexes with acceptable or better quality (DockQ ≥0.23) for 66 of the 112 complexes investigated—25 of which were high quality (DockQ ≥0.8). This is a massive improvement on previous methods with 23 or 47 acceptable models and only four or eight high quality models, when using energy-based docking or interaction templates, respectively. In addition, AlphaFold-Multimer can be used to predict whether a peptide and a protein will interact. At 1% false positives, AlphaFold-Multimer found 26% of the possible interactions with a precision of 85%, the best among the methods benchmarked. However, the most interesting result is the possibility of improving AlphaFold by randomly perturbing the neural network weights to force the network to sample more of the conformational space. This increases the number of acceptable models from 66 to 75 and improves the median DockQ from 0.47 to 0.55 (17%) for first ranked models. The best possible DockQ improves from 0.58 to 0.72 (24%), indicating that selecting the best possible model is still a challenge. This scheme of generating more structures with AlphaFold should be generally useful for many applications involving multiple states, flexible regions, and disorder.

## 1 Introduction

Protein-protein interactions are central to all biological process; knowledge of the molecular details of these interactions is crucial to understanding and altering their function. Up to 40% of protein-protein interactions are considered peptide-protein interactions, with many of these responsible for vital functions such as cell life-cycle regulation (Lee et al., 2019). These are interactions between a protein and a smaller peptide fragment, sometimes referred to as short linear motif (SLiM), which can be part of a disordered region of a larger protein. Because peptide fragments display a high degree of flexibility and are often disordered when unbound, investigating the molecular details of such interactions, and even identifying direct interaction at all, has been difficult both from an experimental and computational point of view. Specialized solutions have been specifically developed to investigate peptide-protein interactions (Petsalaki and Russell, 2008; Alam et al., 2017; Öztürk et al., 2018; Johansson-Åkhe et al., 2021; Lei et al., 2021).

The unprecedented accuracy of AlphaFold (Jumper et al., 2021) and the recent release of its source code have transformed the field of computational and structural biology. It is now possible to achieve highly accurate protein structure prediction on par with experimental accuracy for many proteins—58% of human proteins, for example, can be modeled accurately (Tunyasuvunakool et al., 2021). As a comparison, the previously experimentally determined structures of human proteins had 17% accuracy. This, and the improved methods that will follow in the footsteps of AlphaFold, will have a significant impact not only on structural biology but on the whole field of life-science.

Despite the fact that AlphaFold was trained on monomeric structures, it has demonstrated an impressive ability and stability to allow manipulation of its input to predict protein complexes—for instance, using a 200 residue gap to infer a chain break (Bryant et al., 2022) or using a flexible linker (Ko and Lee, 2021; Tsaban et al., 2022). However, it is clear that, even though the input-adapted versions of AlphaFold performed better than state-of-the-art, the recently released retrained AlphaFold-Multimer system (Evans et al., 2021) successfully predicts the interface (DockQ (Basu and Wallner, 2016a) ≥ 0.23) for 67% of the cases and at high accuracy (DockQ ≥0.8) for 23% of the cases on a set of 4,433 non-redundant interfaces—an improvement over the input-adapted versions of 60% and 12% for the gap and linker, respectively (Evans et al., 2021).

Although AlphaFold expertly implements several state-of-the-art neural network concepts and contributes its own with the Evoformer layers, the common bioinformatics concept of simple increased sampling of the conformational space with more lenient energy terms in search of a global optimum has not been explored. Repeated sampling of predictions with different random seeds or parameters is commonly utilized in bioinformatics methods; examples include Rosetta and ZDOCK (Raveh et al., 2011; Pierce et al., 2014). For many neural networks—AlphaFold included—the random dropout of features inside the network is employed during training of the network to force it to adapt and learn several different ways to solve its target function. Activating dropout layers also at inference would force a network to utilize alternative learned solutions that might be unused or drowned out when the entire network retains all its features, tapping into the alternative learned solutions at the cost of predictive power. This has been previously suggested for introducing and mapping uncertainty while creating a model ensemble with no increase in training time (Gal and Ghahramani, 2016; Lakshminarayanan et al., 2017).

Here, we demonstrate that AlphaFold-Multimer, which has been so successful in the prediction of complexes of globular structures, can also be used to advance the field of peptide-protein complex modeling and interaction prediction. Without any modifications, AlphaFold-Multimer performs much better than state-of-the-art on both peptide-protein complex modeling and interaction prediction. We also show that AlphaFold can be improved by randomly perturbing the neural network weights to force it to sample a larger variety of conformations. By generating a large pool of models and using the excellent scoring function in AlphaFold, it is possible to select much better models than the default setting.

## 2 Materials and methods

### 2.1 Dataset

A test set was constructed for the benchmarking of peptide-protein complex modeling which could also be used for peptide-protein interaction prediction. The samples of observed peptide-protein interaction complexes in the test set were based on the state-of-the art paper for peptide-protein interaction prediction, CAMP, by Lei et al. (2021); it consisted of 262 peptide-protein complexes with experimentally-solved structures in the PDB (Berman et al., 2000). After selecting one complex representative per ECOD (Schaeffer et al., 2017) family, the final redundancy reduced set consisted of 112 peptide-protein complexes.

A negative set was created for peptide-protein interaction prediction in the same manner as in Lei et al. (2021) by randomly pairing 560 peptides and protein receptors from the positive set, creating a negative set five times larger than the positive. Although randomly pairing proteins is no guarantee of lack of interaction, the resulting false negative rate will be statistically insignificant, especially considering that the proteins hail from different species; randomly pairing proteins is, as such, a common practice for constructing negative sets for protein-protein interaction prediction (Guo et al., 2008; Wang et al., 2019).

This scheme results in a dataset which can be used to both evaluate the docking performance on the positive set as well as be used for benchmarking the capacity to predict whether peptide-protein pairs interact or not. Both the positive and negative sets are available as Supplementary Data.

### 2.2 AlphaFold versions

One input-adapted version of AlphaFold monomer and several variants of AlphaFold-Multimer were included in the benchmark, as outlined below:

• *AF-gap*: *This* is an input-adapted version using AlphaFold (Jumper et al., 2021) monomer. The input is adapted by placing a 200-residue gap between the chains and pairing the multiple sequence alignments (MSA) diagonally (Bryant et al., 2022). The models were ranked by the average plDDT (predicted local distance difference test) of the peptide as in Tsaban et al. (2022) and selecting rank 1.
• *AFmulti-*: This is AlphaFold-Multimer version 2.1.0 (Evans et al., 2021). The models were ranked by the *ranking_confidence* score and rank 1 was selected. *ranking_confidence* is a linear combination of the interface score *ipTM* (interface predicted Template Modeling score) and overall structural score *pTM*: 0.8*ipTM* + 0.2*pTM*. AlphaFold multimer was run with one or several options from the list below:
  – *reduced_dbs*: using the reduced database setting.
  – *full_dbs*: using the large sequence databases, BFD, and Uniclust30.
  – *template*: allowing templates as input into AlphaFold. To ensure that the target protein or very close homologs were not used as a template, templates were filtered with BLAST E-values better than 10^–20^ when searching with the target against PDBSEQ or for the peptides > 95% sequence identity over the whole sequence. To allow fair comparison with InterPep2, templates newer than the InterPep2 template library (2019-10-14) were also disallowed.
  – *v2*: AlphaFold-Multimer version 2.2.0 (Evans et al., 2021; Evans et al., 2022) was run rather than 2.1.0.

So, if a variant of AlphaFold-Multimer is denoted as *AFmulti-v2_reduced_dbs_template*, then the AlphaFold-Multimer version 2.2.0 was run with reduced database settings while allowing templates passing the template criteria to be used as input.

The difference between AlphaFold 2.1.0 and 2.2.0 is, according to the AlphaFold authors, that version 2.2.0 has been retrained to produce fewer structural clashes while slightly improving performance. In addition, 2.2.0 generates more structures by default (Evans et al., 2022). Since AlphaFold-Multimer version 2.2.0 generates several structures per model by default, it was also run as restricted to only produce one structure per model so as to enable a fair comparison with AlphaFold-Multimer 2.1.0. In this case, a *1* is added as a suffix to the variant name.

In all cases, AlphaFold was run without the final relaxation step using Amber to save computational time.

#### 2.2.1 AlphaFold database versions

• Uniclust30 version: UniRef30_2021_06
• Uniref90 from 9 August 2021
• Uniprot, TrEMBL + SwissProt, from 3 November 2021.
• BFD database (Steinegger and Söding, 2018), clustering of 2.5 billion sequences from Uniprot/TrEMBL + SwissProt, Metaclust, Soil and Marine Eukaryotic Reference Catalog. Downloaded March 2019. bfd_metaclust_clu_complete_id30_c90_final_seq.sorted_opt_cs219.ffindex MD5 hash: 26d48869efdb50d036e2fb9056a0ae9d
• Mgnify version: 2018_12
• PDB, mmcif and SEQRES, from 3 November 2021 (restrictions when applied at run-time—see above).

### 2.3 InterPep2

InterPep2 (Johansson-Åkhe et al., 2020) is a template-based method for peptide-protein complex structure prediction. It generates multiple peptide conformations and uses TMalign (Zhang and Skolnick, 2005) and InterComp (Mirabello and Wallner, 2018) to find structural templates of interaction, evaluating them by a random forest regressor. The interaction template library was constructed from PDB 2019-10-14. Models for the protein receptors were created using AlphaFold monomer without template information, and InterPep2 was run with standard settings. To avoid using the target protein receptor as a template, any template with a BLAST E-value for the target against PDBSEQ better than 10^–20^ was filtered. This filtered the target protein and very close homologs. Note that it is fine to use homologs and that the main purpose of this filter is to avoid using the target protein. The random forest-predicted suitability of the top-ranking template is used as the score for interaction prediction.

InterPep2 was run without the more computationally intensive refinement protocols, generating only coarse unrefined models. This will result in a lower number of sub-Ångström decoys but will have only minor to no effect on the positioning of the peptide at the correct binding site (Johansson-Åkhe et al., 2020).

### 2.4 PIPER-FlexPepDock (PFPD)

PIPER-FlexPepDock is a state-of-the-art free modeling peptide-protein docking method which combines FFT-based rigid-body docking with high-resolution refinement (Alam et al., 2017). It is based on and builds upon the ClusPro Peptidock method, a previous attempt at rigid-body docking for peptide-proteins (Porter et al., 2017). The PIPER-FlexPepDock protocol generates several possible peptide conformations which are docked on the surface of the receptor protein through PIPER FFT-based docking (Kozakov et al., 2006). The 250 candidates with the lowest score from each of the conformations are all refined using Rosetta FlexPepDock before being clustered (Raveh et al., 2010). The reweighted_sc scoring term was used to rank clustered models.

### 2.5 CABS-dock

CABS-dock utilizes a simulation search with flexible peptide and receptor for peptide-protein docking (Kurcinski et al., 2015; Ciemny et al., 2017). In CABS-dock, coarse-grained, randomized, peptide conformations are randomly distributed around the receptor and then energy-minimized and docked through replica exchange Monte Carlo (REMC) simulations, where the peptide remains completely flexible while the receptor also remains flexible but restrained toward its input state. This results in a wide array of plausible docked conformations, which are then filtered out to 100 conformations per simulation trajectory before repeated k-medoids clustering. The consensus models of the clustering are output as the final predictions. Although the energy function utilized during the REMC scheme could be used to evaluate the final models, this score would be inappropriate as it is used only during sampling; the final models are chosen with regards to clustering.

### 2.6 ZDOCK

ZDOCK is a fast and accurate FFT-based rigid-body docking method which utilizes pairwise statistical potentials; it is a staple method for benchmarking protein–protein docking performance (Pierce and Weng, 2008; Moal et al., 2013; Pierce et al., 2014; Wallner and Mirabello, 2017; Yan and Huang, 2019). In this study, ZDOCK was used to generate 54,000 decoys for each peptide conformation generated in the early steps of the InterPep2 protocol, similar to earlier ZDOCK studies and to ClusPro Peptidock or PIPER-FlexPepDock (Alam et al., 2017; Porter et al., 2017; Wallner and Mirabello, 2017). The best-scoring model was selected as the final prediction.

### 2.7 CAMP

CAMP is a new deep learning-based method that can predict binary peptide-protein interactions (Lei et al., 2021). It uses convolution neural networks and self-attention to extract local and global information using sequence-based input information such as PSSM (position-specific scoring matrix) using three iterations of PSI-BLAST (Altschul et al., 1997) against Uniref90_2019_01 (Suzek et al., 2015) with E = 0.001 for inclusion, predicted disorder, and binding using IUPred2A (Mészáros et al., 2018) and secondary structure prediction using SSPro (Urban et al., 2022). CAMP only predicts if a peptide and a protein will interact, and thus was only included in the interaction benchmark study.

### 2.8 Performance measures

#### 2.8.1 DockQ

The DockQ program was used to assess the quality of docked peptide-protein complexes (Basu and Wallner, 2016a). DockQ assesses the quality of a docked complex with regard to ligand root mean square deviation (LRMSD), the root mean square deviation of the interface residue conformation (iRMSD), and the fraction of native contacts recalled (fnat). The quality is measured on a scale of 0.0–1.0, with 0.25 representing models generally considered acceptably close to native, and 0.8 or above representing models with sub-Ångström quality—see Table 1. DockQ is not a linear measure; while extremely precise sub-Ångström changes in LRMSD may be required to increase DockQ-score from 0.7 to 0.8, when requiring almost perfect structures to achieve a higher DockQ-score, moving a completely flipped peptide toward roughly the correct binding site can be sufficient to raise the DockQ-score from 0.1 to 0.2.

**TABLE 1:** Thresholds for DockQ measure of docked model quality.

DockQ	Model quality
≥0.23	Acceptable
≥0.50	Medium
≥0.80	High

#### 2.8.2 Receiver operating characteristic (ROC)

A ROC curve measures a method’s capacity to discover positive samples versus its number of misclassifications with a sliding score threshold; it plots true positive rate (TPR) versus false positive rate (FPR) for different threshold cutoffs. *TPR* = *Recall* = *TP*/*P* (*TP* = true positives, *P* = total positives in set). *FPR* = *FP*/*N* (*FP* = false positives, *N* = total negatives in set). The ROC area under the curve (AUC) is frequently used as a measure of classification method performance (Johansson-Åkhe et al., 2020; Lei et al., 2021).

#### 2.8.3 Precision, recall, and F1

With an unbalanced dataset, TPR-FPR ROC curves and AUC can be used to benchmark methods against each other; however, it fails to capture the actual absolute performance of the methods, for which precision-recall curves and AUCPR (AUC under such a curve, also referred to as *average precision*) should be used instead (Davis and Goadrich, 2006; Saito and Rehmsmeier, 2015). *Precision* = *TP*/(*TP* + *FP*). *Recall* = *TPR* = *TP*/*P*. F1 is the harmonic mean between precision and recall, defined as *F*1 = (2 ⋅ *Precision* ⋅ *Recall*)/(*Precision* + *Recall*).

## 3 Results and discussion

In this study, the performance of AlphaFold when extended to the peptide-protein docking problem was benchmarked both compared to previously established docking methods and compared to different ways to run AlphaFold. The benchmark was performed on a redundancy-reduced version of a recently published dataset of peptide-protein interactions (Lei et al., 2021) (see Methods section for details). Included in the benchmark were multiple versions of AlphaFold—including *AF-gap*, *AFmulti-reduced_dbs*, *AFmulti-full_dbs*, *AFmulti-reduced_dbs_template*, *AFmulti-full_dbs_template*, *AFmulti-v2_reduced_dbs*, *AFmulti-v2_full_dbs*, *AFmulti-v2_full_dbs1*, *AFmulti-v2_reduced_dbs_template*, and *AFmulti-v2_full_dbs_template*—as well as four existing methods—CABS-dock (Ciemny et al., 2017), PIPER-FlexPepDock (PFPD) (Alam et al., 2017), InterPep2 (Johansson-Åkhe et al., 2020), and ZDOCK (Pierce et al., 2011)—to provide a baseline.

### 3.1 AlphaFold is best with more data and sampling, but worse with templates

**FIGURE 1:**
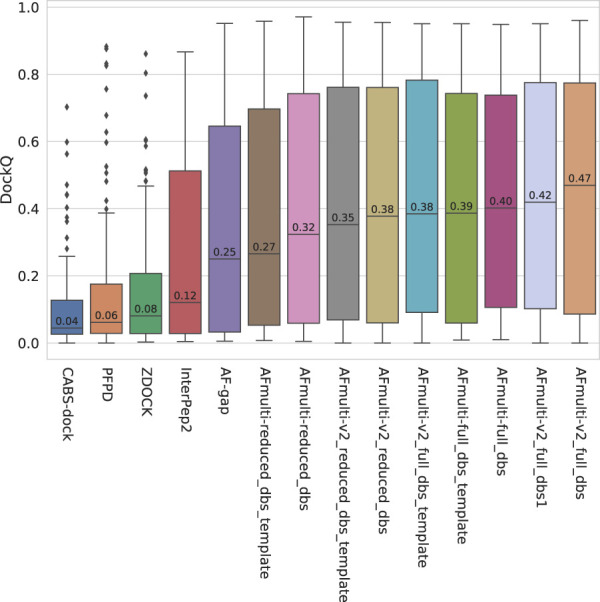
Distribution of model quality as measured by DockQ for the rank 1 models from each method.

The model quality of the top ranked prediction from each method was assessed using DockQ (Basu and Wallner, 2016a). The median DockQ is 0.47 for the best AlphaFold method, AFmulti-v2_full_dbs, compared to 0.12, 0.08, 0.06, and 0.04 for InterPep2, ZDOCK, PFPD, and CABS-dock, respectively—see Figure 1. This clearly indicates that AlphaFold is much better than previously existing methods. AlphaFold-Multimer version 2.2.0 does produce more structures by default, sampling some nearby conformations, but AFmulti-v2_full_dbs1 is still the highest performer of the remaining methods, albeit much closer to AlphaFold-Multimer version 2.1.0 with a median DockQ score of 0.42. AF-gap performs worse than AlphaFold-Multimer versions, with a median DockQ of 0.25, but that is expected since it was not optimized for this task.

Of the different versions of AlphaFold-Multimer tested, the versions with access to the larger databases (full_dbs) are consistently superior. However, running AlphaFold without template information seems better than running it with template information, especially when also run with a reduced database size. Previous work has shown that, although AlphaFold is fully capable of accurately judging the quality of structures derived from template information without any MSA information at all, it cannot efficiently sample the folding landscape without MSAs; if given template information with no MSA information, AlphaFold will essentially copy the template (Roney and Ovchinnikov, 2022). As the peptides are small in size, finding significant sequence matches is difficult and their parts of the paired MSAs often have a low number of effective sequences. This, in combination with the fact that the performance loss when including templates is greater when smaller databases are used for MSA construction, implies that, when run on peptide-protein complexes, the protocol will become over-reliant on using templates to sample starting positions; it ought in such cases to be run without templates.

**FIGURE 2:**
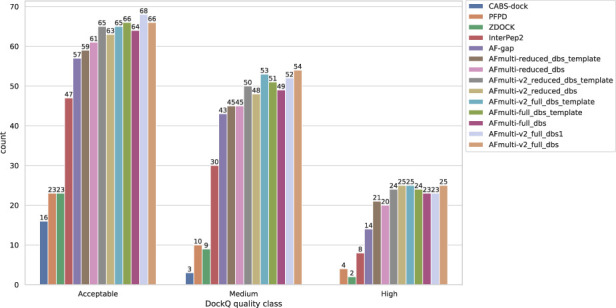
Model quality classes for the different methods. The number of rank 1 models from each method that has a DockQ score above the classification thresholds (Table 1).

By examining the quality in more detail, it can be seen that AlphaFold-Multimer version 2.2.0 (AFmulti-v2_full_dbs) produces more medium quality models than the other methods: 54 compared to only 43 and 30 for AF-gap, and InterPep2, respectively—see Figure 2. Overall, the best AlphaFold-Multimer can predict at least an acceptable model for 68/112 (61%) complexes, while the AlphaFold-Multimer with least information (AFmulti-reduced_dbs) still produces 61/112 (54%) acceptable models.

### 3.2 Forced sampling dramatically improves AlphaFold

Two different methods were employed to force the best versions of AlphaFold-Multimer tested so far to sample more of the conformation space than the standard settings allow. Firstly, the number of recycling steps was incrementally increased from the default three to explore AlphaFold’s own ability to improve its sampling by refining its predictions. Secondly, the dropout layers used during AlphaFold training were activated at inference and the network was run several times with different random seeds, producing many structures for final comparison and evaluation. Similar schemes have been used in the past to create ensemble methods from single trained models and have been used with classifiers to estimate model variance (Gal and Ghahramani, 2016).

**FIGURE 3:**
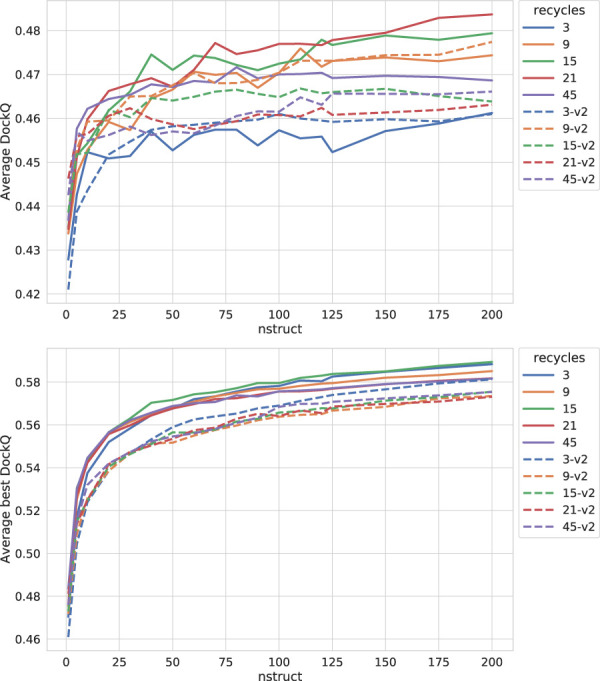
Effects on AlphaFold performance of increasing the number of recycles and allowing more sampling with dropout. The DockQ values reported in the top figure is the median DockQ score of the rank 1 models, and the value reported in the bottom figure is the median of best DockQ score of all models for each target.

As can be seen in Figure 3, both increasing the number of recycles and producing varied samples by running the network several times (nstruct > 1) with dropout active have significant effects on performance, especially when combined. The improvement increases most rapidly for the first 25 structures per network model (5 network models × 25 structures each = 125 total) but, for most settings, there is a small but steady increase in DockQ all the way up to 200 structures (5 × 200 = 1,000 total). We decided to stop at 200 structures in the interest of time, but it is certainly possible that even more structures could improve the method even further. The optimal number of recycles is 21 for v1 and nine for v2. In contrast to dropout, although increasing the recycles does increase performance, the increase does not continually improve with additional recycles. Rather, there are optimal values found within the range sampled rather than at the minimum or maximum values investigated. Increasing the number of recycles permits AlphaFold to spend more cycles refining the same few structures toward its perceived energy minimum. One reason for the slight reduction in performance with recycles increased beyond their optimal values could be that, beyond these many recycles, the refinement of the peptide conformations starts to converge too much and the few output samples will be locked into local energy minima.

**FIGURE 4:**
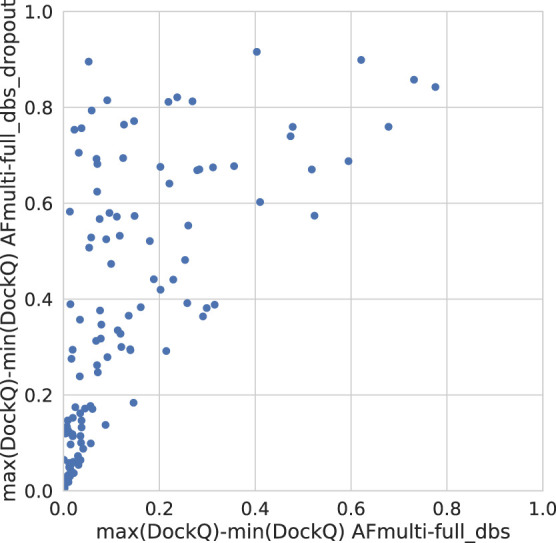
Difference between the maximum and minimum DockQ per target with and without dropout.

**FIGURE 5:**
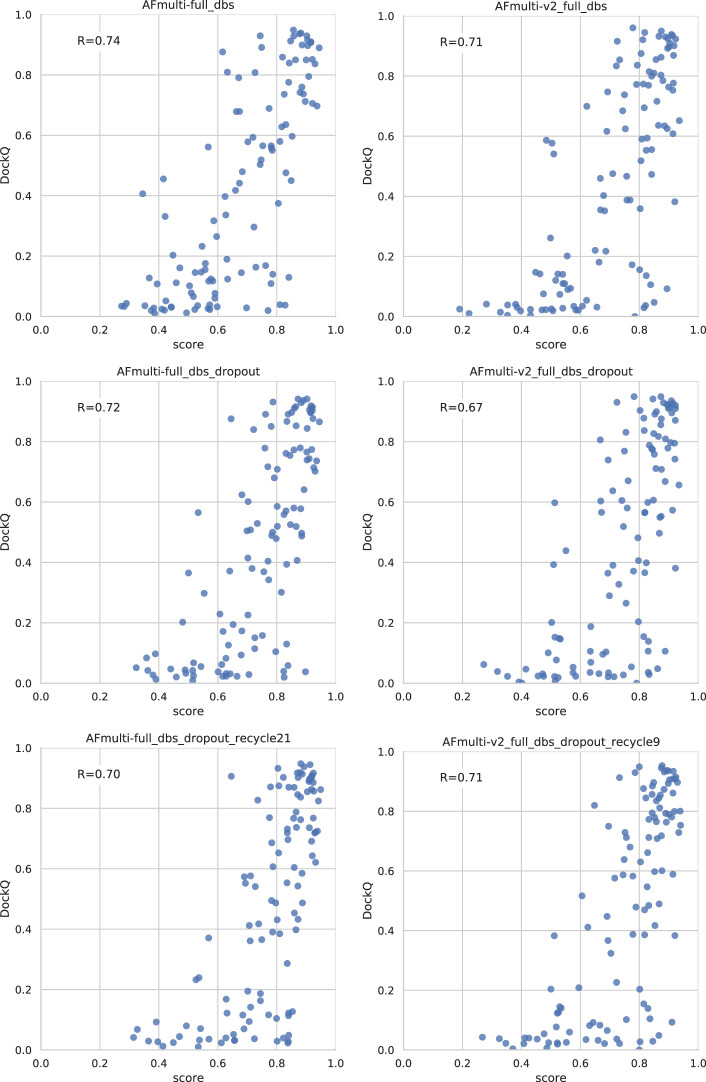
Correlation between method score and DockQ. The score is the *ranking confidence*.

Dropout and increased recycles consistently lead AlphaFold to generate more varied models, including both worse models but also better models than a single run without dropout. The difference between the best and worst DockQ is shown in Figure 4 for each target. In all cases there is a larger spread in favor of dropout. While dropout increases the sampling, the correlation between the self-evaluating score and DockQ decreases somewhat from 0.74 to 0.72 and 0.71 to 0.67 for v1 and v2, respectively (Figure 5). The correlation is still good and the better models can be picked out from the rest so that overall performance increases (Figure 3).

Additionally, combining dropout with increased recycles seems to restore the ranking performance in terms of the correlation of AlphaFold-Multimer version 2.2.0 back up to correlation R of 0.71. For version 2.1.0, the improved method seems to simply generate more high-quality structures without any significant change in the scoring correlation to DockQ (Figure 5). Note, however, that there is still a substantial gap in median DockQ-score for selected models and best models generated (Figure 3), indicating that AlphaFold generates much better models than its scoring function is able to recognize and that there is potential to improve the method by improving the model quality assessment of the generated structures.

For the remainder of this study, AlphaFold-Multimer run with dropout produces 200 structures per neural network model (5 × 200 = 1,000 structures in total). Whenever AlphaFold-Multimer-v1 is referenced as being run with additional recycles, it is run with 21 recycles unless otherwise specified. Similarly, AlphaFold-Multimer-v2 is run with nine recycles. Note that the base behavior of AlphaFold is to run three recycles unless otherwise specified. These specific versions of AlphaFold-Multimer were selected based on the median DockQ score of their predictions (Figure 3).

### 3.3 Greater improvements to AlphaFold by selective dropout

Even though running AlphaFold-Multimer with dropout active at inference improved the quality of final predicted docked peptide complexes, the correlation between AlphaFold predicted confidence and DockQ became worse (Figure 5). This is unsurprising, as dropout is not only applied to the evoformer layers of the network but also to the structure module responsible for translating the latent representation of the evoformer layers into the final protein structure, as well as for creating the final predicted confidence score. Roney and Ovchinnikov (2022) theorized that the purpose of the evoformer layers is to provide an initial low-energy guess of the protein structure, while the structure module refines this guess. If this theory holds, then including dropout at inference in the early layers of the AlphaFold network should be enough to introduce variance in final predictions and explore conformational space, while including dropout in the structure module might be used for uncertainty assessment but would have detrimental effects on final predicted complexes, particularly the score.

To test this hypothesis, AlphaFold-Multimer versions 2.1.0 and 2.2.0 were run again on the entire dataset, using the best combination of hyper-parameters such as number of recycles and dropout seen in previous tests. This time, dropout was applied selectively to all parts of the network except the structure module, in the hope that the correlation between confidence score and DockQ would be retained in relation to running without dropout, thereby also improving the selection between generated structures and thus improving AlphaFold-Multimer for peptides overall. These versions are denoted with *dropout_noSM* for dropout but no dropout in the structure module (SM).

Table 2 shows that, for AlphaFold-Multimer version 2.1.0, 3 recycles, this hypothesis holds true and correlation between predicted score and DockQ is restored while the overall model quality is improved with no dropout in the structure module (noSM); correlation/median DockQ for dropout 0.72/0.50 vs. 0.75/0.53 for dropout + noSM. However, for the optimal number of recycles (21), no dropout in the structural model seems to have no effect on the correlation and median DockQ.

**TABLE 2:** Summary of results for different methods and settings. *Corr*. is the correlation between reported scores and DockQ, *Median DockQ* is the median DockQ when selecting rank 1 for each target. The best values for a particular group are highlighted in bold. *CABS-dock does not output any score: it only returns ranked models.

Predictor	Version	Recycles	Dropout	Corr.	Median DockQ
CABS-dock	—	—	—	-*	0.05
PIPER-FlexPepDock	—	—	—	0.16	0.06
ZDOCK	—	—	—	0.07	0.08
InterPep2	—	—	—	**0.76**	0.12
AF-gap	2.0.0	3	no	0.42	0.25
AlphaFold-Multimer	2.1.0	3	no	0.74	0.40
	2.1.0	3	yes	0.72	0.50
	2.1.0	3	yes, noSM	**0.75**	0.53
	2.1.0	21	no	**0.75**	0.43
	2.1.0	21	yes	0.70	0.54
	2.1.0	21	yes, noSM	0.70	**0.55**
AlphaFold-Multimer	2.2.0	3	no	**0.71**	0.47
	2.2.0	3	yes	0.67	0.51
	2.2.0	3	yes, noSM	0.67	0.50
	2.2.0	9	no	0.68	0.49
	2.2.0	9	yes	**0.71**	**0.53**
	2.2.0	9	yes, noSM	**0.71**	0.50

The same table also shows that AlphaFold-Multimer version 2.2.0 has no benefit from no dropout in the structure module at all: the performance drops from 0.51 to 0.50 and 0.53 to 0.50 for three and nine recycles, respectively, and the correlation does not change. We speculate that the reason version 2.1.0 seems to be more affected by increased sampling compared to 2.2.0 may be the increased loss term for clashing models that was introduced in 2.2.0 (Evans et al., 2021). Of course, the final model should not contain severe clashes but, during sampling, it might be advantageous to allow for some structural overlap to enable the method to explore various predictions, similar to a soft repulsive energy term that is often used during refinement (Raveh et al., 2011; Nivón et al., 2013). This might also explain why version 2.2.0 requires fewer recycles to reach optimal performance than version 2.1.0. As the focus on fine-grained refinement performance is greater, the number of refinement recycles required is fewer.

### 3.4 Complementary performance of AlphaFold multimer v1 and v2

While both AlphaFold-Multimer versions 2.1.0 and 2.2.0 show higher performance with the increased sampling through dropout—with 2.1.0 seeing a larger improvement but 2.2.0 already starting at a higher performance level—the two versions show differences in their overall behavior. Version 2.1.0 performs better at high numbers of recycles and when the structure module is not subjected to dropout. Version 2.2.0, on the other hand, seems to require dropout in all layers to perform optimally, and a generally lower number of recycles is better.

**FIGURE 6:**
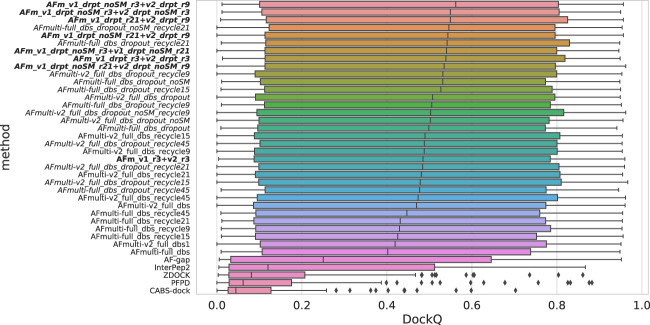
Distributions of DockQ scores for final selected predictions for each target from each method. Variations on AlphaFold-Multimer version 2.2.0 are marked in italics while combinations of versions 2.1.0 and 2.2.0 are marked in bold with *full_dbs* removed since it is used in all methods. The abbreviations *drpt* and *r* stand for *dropout* and *recycles*, respectively. The color is a gradient based on the rank of the median DockQ.

**FIGURE 7:**
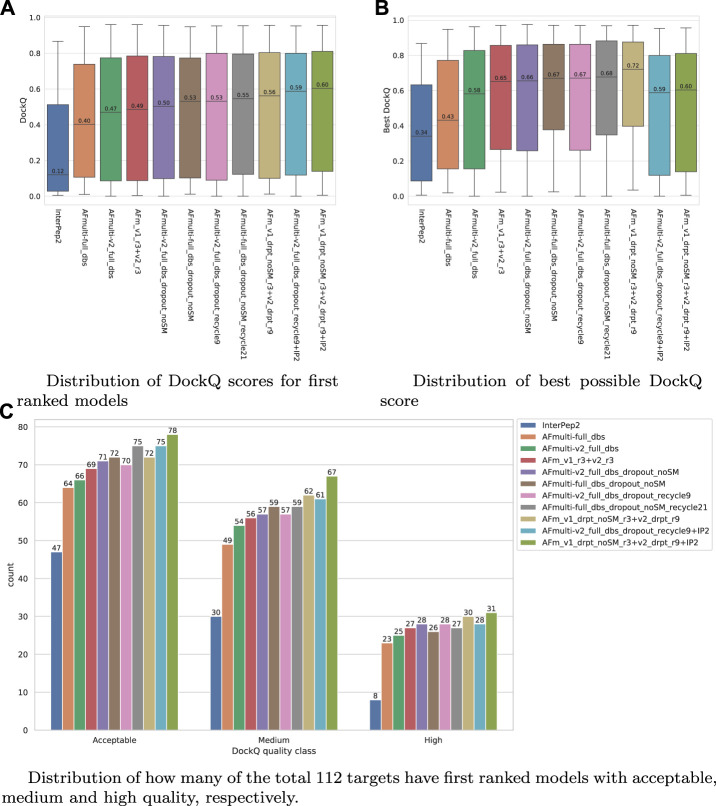
Performance of different AlphaFold-Multimer versions; InterPep2 is included as a reference to the previous methods. **(A)** Distribution of DockQ scores for first ranked models. **(B)** Distribution of best possible DockQ score. **(C)** Distribution of how many of the total 112 targets have first-ranked models with acceptable, medium, and high quality, respectively.

Since both versions produce predictions in the same predicted score range and both correlate their scores well with DockQ, it is simple to construct combination predictors by allowing the different versions to generate 100 structures each and then selecting the structure with the overall highest predicted score (200 structures in total, for fair comparison with other versions). Such different combinations can be compared to the versions of AlphaFold-Multimer investigated so far in Figure 6. Indeed, such a simple combination manages to increase performance even further than the increased sampling alone, raising it from median DockQ scores of 0.53 or 0.55 to 0.56 for the best combination. The best combination (AFm_v1_drpt_noSM_r3 + v2_drpt_r9) consists of the versions with best correlations between predicted score and DockQ score, and AFmulti-full_dbs_dropout_noSM and AFmulti-v2_full_dbs_dropout_recycle9; even though AFmulti-full_dbs_dropout_noSM_recycle21 is the version of AlphaFold-Multimer 2.1.0 with the best median DockQ, it is not part of the best combination. At this point, it seems as though the correlation between ranking score and DockQ is more important than the median model quality of the models generated, as models are already generated with a far higher quality than that selected (Figure 7B).

#### 3.4.1 Complementary performance of AlphaFold-Multimer and InterPep2

While the performance of AlphaFold-Multimer, especially with the improvements described in this study, is far above the template-based method InterPep2, both AlphaFold-Multimer and InterPep2 have scores that correlate well with DockQ. In addition, AlphaFold-Multimer does not use its templates for the multimer interaction but only for folding the individual chains. Thus, if AlphaFold-Multimer is uncertain in its prediction, it is possible that a template for the interaction found by InterPep2 can be used. A combination method can be constructed, using the scores for confident predictions by AlphaFold-Multimer (ranking_confidence > 0.70) (Evans et al., 2021) and InterPep2 (ip2_score > 0.40) (Johansson-Åkhe et al., 2020), to select models in the following priority: 1) AlphaFold-Multimer model if score is confident; 2) InterPep2 model if score is confident; otherwise 3) AlphaFold-Multimer model. This selection scheme does not differ from previous choices between using template-based or template-free modeling. The only difference is that AlphaFold has changed the playing field and template-free modeling with AlphaFold is now potentially better than template-based if none of the methods produce models with confident scores.

The results for the AlphaFold + InterPep2 combo predictor for peptide-protein docking can be found in Figure 7. The performance increase from including InterPep2 is surprisingly substantial, raising the median DockQ from 0.562 to 0.604, even though InterPep2 by itself produces models with a much lower median DockQ than all AlphaFold versions tested. This probably stems from the high correlation between AlphaFold and InterPep2 predicted scores and DockQ score. Indeed, when looking at the best models sampled for the best combination methods, they all reach median DockQ scores in excess of 0.7. However, the final selected models have median DockQ scores of only up to 0.6 in the case of the very best combination. Conformations of higher quality are obviously sampled but are not selected by the predicted score, indicating yet again that, when running AlphaFold with dropout, a remaining challenge is model ranking.

### 3.5 Trends in factors contributing to the performance of AlphaFold

To analyze potential trends in factors that contribute to the performance of AlphaFold, we correlated the performance with physiochemical and evolutionary factors that might influence the results—see Supplementary Figure S1. Apart from the factors that can be calculated from AlphaFold input data, such as protein lengths and MSA depths, an updated version of ProQDock (Basu and Wallner, 2016b) was used to calculate the shape complementarity (Sc) (Lawrence and Colman, 1993) and electrostatic complementarity (EC) (McCoy et al., 1997) of native and modelled peptide-protein interfaces; DISOPRED (Jones and Cozzetto, 2014) was used to estimate the amount of disorder in the peptide in isolation and Proteus (Basu et al., 2017) to predict disorder-to-order transitions for the peptide residues upon binding.

Sc for the native peptide-protein interfaces follows a skewed Gaussian distribution where the majority of the targets have Sc > 0.6, while the EC for the native interfaces follows a Gaussian centered at 0—see Supplementary Figure S1. This indicates that surface complementarity is an important factor for native peptide-protein binding and that many native interactions are not driven by favorable electrostatic interactions. A comparison of the EC distribution for the native and AlphaFold models (denoted EC and EC_v1 in the figure, respectively) reveals that they are significantly different (*p*-value < 1-e7) and that the models lack many high EC peptide-protein interfaces. A potential reason for this could be that the models are not properly refined because the AlphaFold models evaluated here are taken straight from the neural network before the relaxation step using Amber.

As in previous reports (Tsaban et al., 2022), we could not observe any correlation between model quality and receptor or peptide length. Neither did the number of effective sequences in the MSA have any general impact; there are some poor predictions with relatively shallow MSAs N_*eff*_ < 100, as expected, but there are also some poor predictions with N_*eff*_ > 1,000—see Supplementary Figure S1. Even though the difference in average DockQ score for these two groups is quite large—0.19 vs. 0.42—the small sample size makes the difference insignificant (*p*-value > 0.1).

Only 16% of the peptides in the test set are predicted to be almost completely disordered when unbound (more than 75% of the residues predicted as disordered). The average DockQ scores for complexes with peptides predicted as disordered when unbound are lower than the average DockQ score for the targets with peptides predicted as ordered when unbound—0.32 vs. 0.43—but the difference is not significant (*p*-value > 0.19). Quite a large percentage—58%—of the targets are predicted to undergo a disorder-to-order transition to accommodate the binding (peptides with > 25% of its residues predicted by Proteus to undergo disorder-to-order transitioning upon binding). Surprisingly, these targets have a significantly larger average DockQ score compared to the targets that are not predicted to fold upon binding—0.48 vs. 0.33 (*p*-value < 0.02).

### 3.6 AlphaFold can be used for interaction prediction

Since the AlphaFold-Multimer predicted score correlates well with the DockQ score of the predicted complexes and since the median DockQ of predicted complexes for true interactions is well above the Acceptable DockQ cutoff, it might be possible to use AlphaFold as an interaction predictor.

**FIGURE 8:**
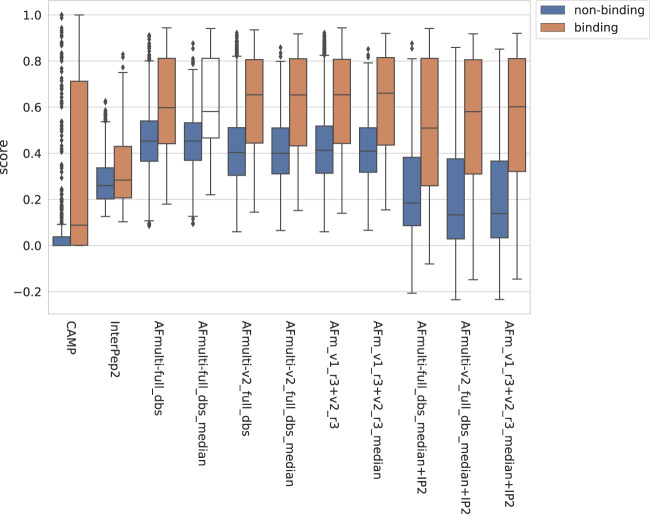
Distribution of scores reported by the method for non-binding and binding peptide-protein pairs, respectively, from different versions of AlphaFold, with CAMP and InterPep2 as reference previous methods.

The positive set used to assess the complex model quality above was merged with the negative set containing peptide-protein pairs that do not bind (see Materials and Methods). For AlphaFold-Multimer, additional methods based on the median score of the generated models rather than the best score were added, suffixed with *_median*. The rationale for using the median is that it should be more stable than the best score, which is based on only one model. The scores for binding vs. non-binding pairs are shown in Figure 8. In general, pairs that bind are scored higher than non-binders across all methods.

**FIGURE 9:**
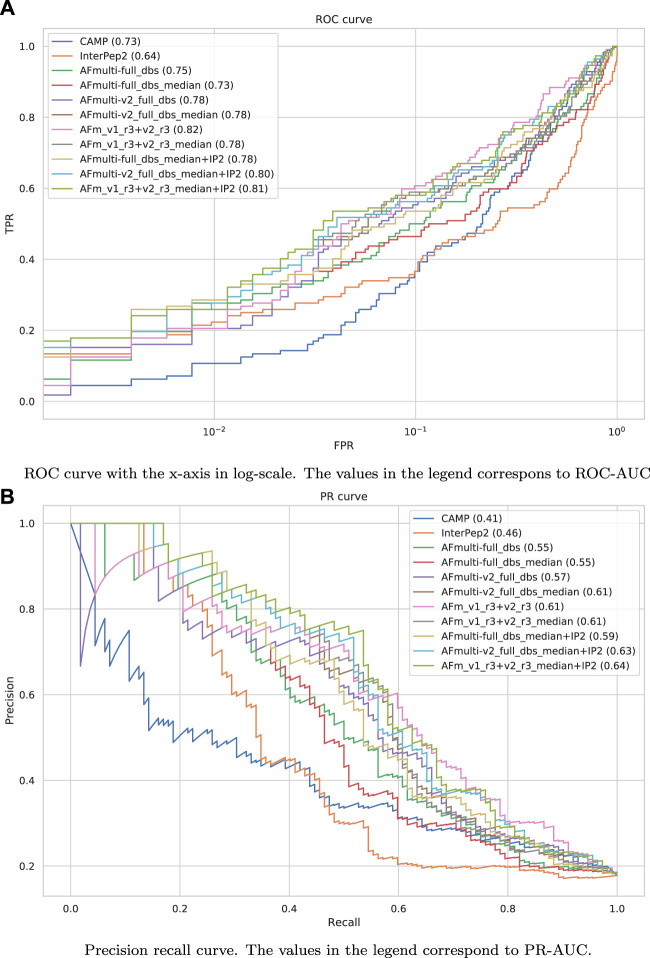
Performance measures for peptide-protein interaction prediction. **(A)** ROC curve with the *x*-axis in log-scale. The values in the legend correspond to ROC-AUC. **(B)** Precision recall curve. The values in the legend correspond to PR-AUC.

The precision-recall-AUC (PR-AUC) is 0.64 for AFm_v1_r3+_v2_r3_median + IP2 compared to 0.61 for AFm_v1_r3+_v2_r3_median and the best single AlphaFold-Multimer version (AFmulti-v2_full_dbs_median). Using the median score improves the PR-AUC from 0.57 to 0.61 for AFmulti-v2_full_dbs, indicating that picking the median predicted score rather than the highest predicted score seems to yield slightly better performance (Figure 9B).

**FIGURE 10:**
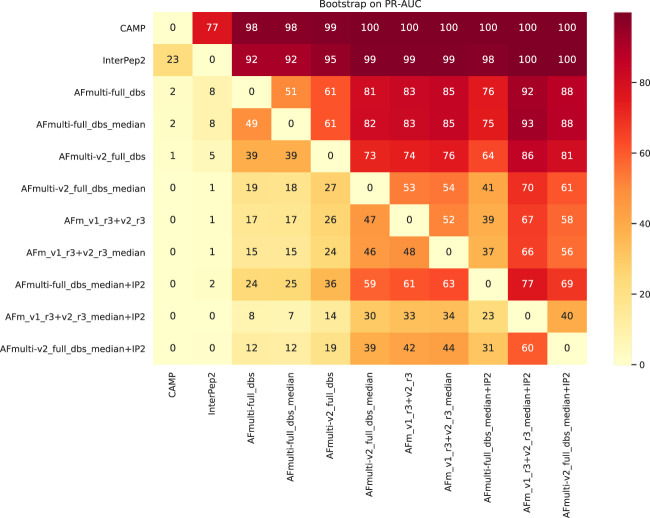
Pairwise bootstrap values on PR-AUC, corresponding to the percentage of times the method on the *X*-axis has a *higher* PR-AUC than the method on the *Y*-axis when sampling the data with replacement (*n* = 10,000).

To assess the significance of the PR-AUC values, a bootstrap procedure in which targets were selected with replacement and the PR-AUC was recalculated was used. Methods were compared pairwise and the process was repeated 10,000 times. The bootstrap values (percentage of times in which one method has a PR-AUC better than the other) are presented in Figure 10. The bootstrap value for AFm_v1_r3+_v2_r3_median + IP2 (PR-AUC = 0.64) against AFm_v1_r3+_v2_r3_median (PR-AUC = 0.61) is 67, and 70 against the best single AFmulti-v2_full_dbs_median (PR-AUC = 0.61). Using the median for AFmulti-v2_full_dbs produces a higher PR-AUC in 73% of the cases. This shows that, although differences in PR-AUC seem incremental the improvements investigated here cause the improved methods to produce higher quality models a clear majority of the time (≥60% for the best method investigated compared to all others).

Performance for interaction prediction needs to be assessed at low FPR to enable all-vs-all comparisons with high confidence predictions without too many false positives. At FPR = 0.01, the best methods recall around 0.26–0.29 (TPR) of the positive examples with a precision of 0.82–0.85—see Figure 9A and Table 3. The corresponding score thresholds for the methods are 0.78, 0.50, and 0.78 for AlphaFold-Multimer (v1 and v2), InterPep2, and AFm_v1_r3+_v2_r3_median + InterPep2, respectively—see Supplementary Figure S2. For AlphaFold, there is an almost a 1 to 1 correspondence with the precision of the predictions and the score threshold—see Supplementary Figure S2A—indicating that that score is a relatively good predictor of the probability of interaction.

**TABLE 3:** Summary of interaction prediction results measured by precision, recall at FPR = 0.01, and FPR = 0.1.

Method	FPR = 0.01		FPR = 0.1	
	precision	recall	precision	recall
CAMP	0.67	0.07	0.43	0.35
InterPep2	0.83	0.21	0.44	0.36
AFmulti-full_dbs	0.85	0.20	0.53	0.48
AFmulti-full_dbs_median	0.84	0.29	0.49	0.46
AFmulti-v2_full_dbs	0.82	0.16	0.54	0.55
AFmulti-v2_full_dbs_median	0.85	0.26	0.55	0.58
AFm_v1_r3+v2_r3	0.79	0.21	0.57	0.60
AFm_v1_r3+v2_r3_median	0.83	0.26	0.58	0.58
AFmulti-full_dbs_median + IP2	0.84	0.29	0.56	0.53
AFmulti-v2_full_dbs_median + IP2	0.85	0.26	0.52	0.56
AFm_v1_r3+v2_r3_median + IP2	0.83	0.26	0.53	0.58

### 3.7 Example—Improvements for AlphaFold

**FIGURE 11:**
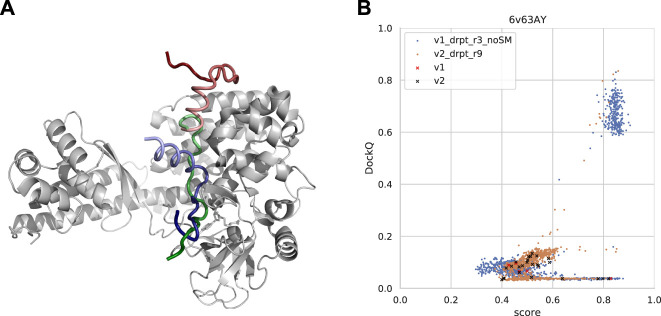
**(A)** An example from the test set where both forced sampling through dropout and combination with the template-based method, InterPep2, can improve the prediction. The predictions are of a cytoplasmic actin peptide binding to actin-histidine N-methyltranferase (PDB ID 6v63). The top predictions by AlphaFold-Multimer positions the very end of the C-terminal in the correct site (top1 prediction in red and native peptide colored green) but positions the peptide in the opposite direction of the actual fold, leading to most of it binding outside the pocket. The prediction by InterPep2 (blue) is coarse with some minor clashes but has positioned the peptide inside the correct binding site. Note how both AlphaFold and InterPep2 have adopted similar folds for the peptide. **(B)** Scatterplot of AlphaFold-Multimer predicted score versus DockQ for all models generated for the 6v63 complex. When dropout at inference forces increased sampling, some conformations are sampled in the correct binding pose.

Cases where AlphaFold failed were examined to understand why AlphaFold fails for certain samples as well as why forced sampling through dropout and the template-based method InterPep2 can help with prediction quality in these cases. One such example can be seen in Figure 11A, where AlphaFold has positioned part of the peptide correctly but has flipped the orientation of the peptide and positioned the rest of it outside its binding pocket, as if to continue the peptide chain in that direction. In fact, in 50% of cases where the AFmulti-v2_full_dbs_dropout_recycle9+IP2 method produced an improved model of at least acceptable quality while AFmulti-v2_full_dbs_dropout_recycle9 alone failed, the AlphaFold model positioned the peptide close to or at the correct binding site but in the wrong direction.

In this particular case, increased sampling through forced dropout with AlphaFold-Multimer version 2.1.0 could also result in the correct conformation being sampled and selected, as AlphaFold samples were more nearby the conformational space, including the reverse direction of the peptide (and some conformations half-way rotated). In fact, the increased sampling for version 2.2.0 also samples a few high-DockQ conformations, although none of these are scored high enough to be selected (Figure 11B).

In 33% of improved cases, AlphaFold positioned the peptide at the binding site of a non-peptide binding partner, either for a co-factor or another protein chain (dimerization site, for instance). AlphaFold seems able to model the receptor protein regardless of the presence of any other binding chains or co-factors (Jumper et al., 2021; Tunyasuvunakool et al., 2021). However, as is evident from these examples, the absence of other binding partners and co-factors from the AlphaFold-Multimer modeling seems to result in a negative bias on the docking performance. Co-factors and other binders being absent from a complex seems to have less effect on the performance of the purely template-based method InterPep2, perhaps explaining in part why it is a good complement to AlphaFold-Multimer.

## 4 Conclusion

We have shown that AlphaFold-Multimer achieves state-of-the-art performance in both peptide-protein docking and peptide-protein interaction prediction without modification. Most interesting, however, is the discovery that forcing increased sampling of the conformational space by increasing the number of recycles of the final layers of the network or adding dropout at inference and running the protocol several times can significantly improve the performance of AlphaFold.

The improvement median DockQ score when using the improved sampling strategy is dramatic, from 0.40/0.42 to 0.55/0.53 for AlphaFold-Multimer versions v1 and v2, respectively. By combining both versions v1 and v2, it possible to raise the median DockQ to 0.56; adding a template-based method (InterPep2) for complexes where AlphaFold produces low confidence predictions raises the median DockQ as high as 0.60.

These results reinforce the previous findings that AlphaFold is well-suited for the peptide-protein docking problem which requires a wider sampling of conformations, especially with our modifications to improve sampling. It should be noted that the improved sampling protocol presented here is not limited to the peptide-protein docking problem and should be useful in many AlphaFold applications. Examples are, for investigating multiple stable conformations, for larger and more difficult targets, or for larger assemblies. Additionally, more variations in the application of dropout at inference could be investigated, such as different rates of dropout.

Finally, while the improvements presented here are significant, there is still a large gap between the quality of the best model generated and the one ranked highest by AlphaFold’s predicted score. As such, protein model quality assessment remains an important field of research in protein structure prediction with AlphaFold.

## Data Availability

The original contributions presented in the study are included in the article/Supplementary Material, and further inquiries can be directed to the corresponding author.

## References

[B1] Alam N. Goldstein O. Xia B. Porter K. A. Kozakov D. Schueler-Furman O. (2017). High-resolution global peptide-protein docking using fragments-based PIPER-FlexPepDock. PLoS Comput. Biol. 13 (12), e1005905. 10.1371/journal.pcbi.1005905 29281622 PMC5760072

[B2] Altschul S. F. Madden T. L. Schäffer A. A. Zhang J. Zhang Z. Miller W. (1997). Gapped BLAST and PSI-BLAST: A new generation of protein database search programs. Nucleic Acids Res. 25 (17), 3389–3402. 10.1093/nar/25.17.3389 9254694 PMC146917

[B3] Basu S. Söderquist F. Wallner B. (2017). Proteus: A random forest classifier to predict disorder-to-order transitioning binding regions in intrinsically disordered proteins. J. Comput. Aided. Mol. Des. 31 (5), 453–466. 10.1007/s10822-017-0020-y 28365882 PMC5429364

[B4] Basu S. Wallner B. (2016a). Dockq: A quality measure for protein-protein docking models. PloS one 11 (8), e0161879. 10.1371/journal.pone.0161879 27560519 PMC4999177

[B5] Basu S. Wallner B. (2016b). Finding correct protein–protein docking models using proqdock. Bioinformatics 32 (12), i262–i270. 10.1093/bioinformatics/btw257 27307625 PMC4908341

[B6] Berman H. M. Westbrook J. Feng Z. Gilliland G. Bhat T. N. Weissig H. (2000). The protein data bank. Nucleic Acids Res. 28 (1), 235–242. 10.1093/nar/28.1.235 10592235 PMC102472

[B7] Bryant P. Pozzati G. Elofsson A. (2022). Improved prediction of protein-protein interactions using AlphaFold2. Nature Communications 13, 1265. bioRxiv. 10.1038/s41467-022-28865-w PMC8913741 35273146

[B8] Ciemny M. P. Kurcinski M. Kozak K. J. Kolinski A. Kmiecik S. (2017). “Highly flexible protein-peptide docking using cabs-dock,” in Modeling peptide-protein interactions (Springer), 69–94. 10.1007/978-1-4939-6798-8_6 28236234

[B9] Davis J. Goadrich M. (2006). “The relationship between precision-recall and roc curves,” in Proceedings of the 23rd international conference on Machine learning, 233–240.

[B10] Evans R. O’Neill M. Pritzel A. Antropova N. Senior A. Green T. (2022). Alphafold/v2.2.0. Available at: https://github.com/deepmind/alphafold/tree/v2.2.0 . 10.1101/2021.10.04.463034

[B11] Evans R. O’Neill M. Pritzel A. Antropova N. Senior A. Green T. (2021). Protein complex prediction with AlphaFold-Multimer. bioRxiv.

[B12] Gal Y. Ghahramani Z. (2016). “Dropout as a bayesian approximation: Representing model uncertainty in deep learning,” in International conference on machine learning (PMLR), 1050–1059.

[B13] Guo Y. Yu L. Wen Z. Li M. (2008). Using support vector machine combined with auto covariance to predict protein–protein interactions from protein sequences. Nucleic acids Res. 36 (9), 3025–3030. 10.1093/nar/gkn159 18390576 PMC2396404

[B14] Johansson-Åkhe I. Mirabello C. Wallner B. (2020). InterPep2: Global peptide-protein docking using interaction surface templates. Bioinformatics 36, 2458. 10.1093/bioinformatics/btaa005 31917413 PMC7178396

[B15] Johansson-Åkhe I. Mirabello C. Wallner B. (2021). Interpeprank: Assessment of docked peptide conformations by a deep graph network. Front. Bioinform. 1, 60. 10.3389/fbinf.2021.763102 PMC9581042 36303778

[B16] Jones D. T. Cozzetto D. (2014). DISOPRED3: Precise disordered region predictions with annotated protein-binding activity. Bioinforma. Oxf. Engl. 31 (6), 857. 10.1093/bioinformatics/btu744 PMC4380029 25391399

[B17] Jumper J. Evans R. Pritzel A. Green T. Figurnov M. Ronneberger O. (2021). Highly accurate protein structure prediction with AlphaFold. Nature 596, 583–589. 10.1038/s41586-021-03819-2 34265844 PMC8371605

[B18] Ko J. Lee J. (2021). Can AlphaFold2 predict protein-peptide complex structures accurately? bioRxiv. 10.1101/2021.07.27.453972

[B19] Kozakov D. Brenke R. Comeau S. R. Vajda S. (2006). Piper: An fft-based protein docking program with pairwise potentials. Proteins. 65 (2), 392–406. 10.1002/prot.21117 16933295

[B20] Kurcinski M. Jamroz M. Blaszczyk M. Kolinski A. Kmiecik S. (2015). CABS-dock web server for the flexible docking of peptides to proteins without prior knowledge of the binding site. Nucleic Acids Res. 43 (W1), W419–W424. 10.1093/nar/gkv456 25943545 PMC4489223

[B21] Lakshminarayanan B. Pritzel A. Blundell C. (2017). Simple and scalable predictive uncertainty estimation using deep ensembles. Adv. neural Inf. Process. Syst. 30.

[B22] Lawrence M. C. Colman P. M. (1993). Shape complementarity at protein/protein interfaces. J. Mol. Biol. 234 (4), 946–950. 10.1006/jmbi.1993.1648 8263940

[B23] Lee A. C.-L. Harris J. L. Khanna K. K. Hong J.-H. (2019). A comprehensive review on current advances in peptide drug development and design. Int. J. Mol. Sci. 20 (10), 2383. 10.3390/ijms20102383 PMC6566176 31091705

[B24] Lei Y. Li S. Liu Z. Wan F. Tian T. Li S. (2021). A deep-learning framework for multi-level peptide–protein interaction prediction. Nat. Commun. 12 (1), 5465. 10.1038/s41467-021-25772-4 34526500 PMC8443569

[B25] McCoy A. J. Epa V. C. Colman P. M. (1997). Electrostatic complementarity at protein/protein interfaces 1 1Edited by B. Honig. J. Mol. Biol. 268 (2), 570–584. 10.1006/jmbi.1997.0987 9159491

[B26] Mészáros B. Erdos G. Dosztányi Z. (2018). IUPred2A: Context-dependent prediction of protein disorder as a function of redox state and protein binding. Nucleic acids Res. 46 (W1), W329–W337. 10.1093/nar/gky384 29860432 PMC6030935

[B27] Mirabello C. Wallner B. (2018). Topology independent structural matching discovers novel templates for protein interfaces. Bioinformatics 34 (17), i787–i794. 10.1093/bioinformatics/bty587 30423106

[B28] Moal I. H. Torchala M. Bates P. A. Fernández-Recio J. (2013). The scoring of poses in protein-protein docking: Current capabilities and future directions. BMC Bioinforma. 14 (1), 286. 10.1186/1471-2105-14-286 PMC3850738 24079540

[B29] Nivón L. G. Moretti R. Baker D. (2013). A pareto-optimal refinement method for protein design scaffolds. PloS one 8 (4), e59004. 10.1371/journal.pone.0059004 23565140 PMC3614904

[B30] Öztürk H. Özgür A. Ozkirimli E. (2018). Deepdta: Deep drug–target binding affinity prediction. Bioinformatics 34 (17), i821–i829. 10.1093/bioinformatics/bty593 30423097 PMC6129291

[B31] Petsalaki E. Russell R. B. (2008). Peptide-mediated interactions in biological systems: New discoveries and applications. Curr. Opin. Biotechnol. 19 (4), 344–350. 10.1016/j.copbio.2008.06.004 18602004

[B32] Pierce B. G. Hourai Y. Weng Z. (2011). Accelerating protein docking in ZDOCK using an advanced 3D convolution library. PloS one 6 (9), e24657. 10.1371/journal.pone.0024657 21949741 PMC3176283

[B33] Pierce B. G. Wiehe K. Hwang H. Kim B.-H. Vreven T. Weng Z. (2014). ZDOCK server: Interactive docking prediction of protein–protein complexes and symmetric multimers. Bioinformatics 30 (12), 1771–1773. 10.1093/bioinformatics/btu097 24532726 PMC4058926

[B34] Pierce B. Weng Z. (2008). A combination of rescoring and refinement significantly improves protein docking performance. Proteins. 72 (1), 270–279. 10.1002/prot.21920 18214977 PMC2696687

[B35] Porter K. A. Xia B. Beglov D. Bohnuud T. Alam N. Schueler-Furman O. (2017). Cluspro peptidock: Efficient global docking of peptide recognition motifs using fft. Bioinformatics 33 (20), 3299–3301. 10.1093/bioinformatics/btx216 28430871 PMC5860028

[B36] Raveh B. London N. Schueler-Furman O. (2010). Sub-angstrom modeling of complexes between flexible peptides and globular proteins. Proteins. 78 (9), 2029–2040. 10.1002/prot.22716 20455260

[B37] Raveh B. London N. Zimmerman L. Schueler-Furman O. (2011). Rosetta flexpepdock *ab-initio*: Simultaneous folding, docking and refinement of peptides onto their receptors. PloS One 6 (4), e18934. 10.1371/journal.pone.0018934 21572516 PMC3084719

[B38] Roney J. P. Ovchinnikov S. (2022). State-of-the-art estimation of protein model accuracy using alphafold. bioRxiv. 10.1101/2022.03.11.484043 PMC12178128 36563190

[B39] Saito T. Rehmsmeier M. (2015). The precision-recall plot is more informative than the roc plot when evaluating binary classifiers on imbalanced datasets. PloS one 10 (3), e0118432. 10.1371/journal.pone.0118432 25738806 PMC4349800

[B40] Schaeffer R. D. Liao Y. Cheng H. Grishin N. V. (2017). Ecod: New developments in the evolutionary classification of domains. Nucleic Acids Res. 45 (D1), D296–D302. 10.1093/nar/gkw1137 27899594 PMC5210594

[B41] Steinegger M. Söding J. (2018). Clustering huge protein sequence sets in linear time. Nat. Commun. 9 (1), 2542. 10.1038/s41467-018-04964-5 29959318 PMC6026198

[B42] Suzek B. E. Wang Y. Huang H. McGarvey P. B. Wu C. H. Consortium U. (2015). UniRef clusters: A comprehensive and scalable alternative for improving sequence similarity searches. Bioinformatics 31 (6), 926–932. 10.1093/bioinformatics/btu739 25398609 PMC4375400

[B43] Tsaban T. Varga J. K. Avraham O. Ben-Aharon Z. Khramushin A. Schueler-Furman O. (2022). Harnessing protein folding neural networks for peptide–protein docking. Nat. Commun. 13 (1), 176–212. 10.1038/s41467-021-27838-9 35013344 PMC8748686

[B44] Tunyasuvunakool K. Adler J. Wu Z. Green T. Zielinski M. Žídek A. (2021). Highly accurate protein structure prediction for the human proteome. Nature 596 (7873), 590–596. 10.1038/s41586-021-03828-1 34293799 PMC8387240

[B45] Urban G. Magnan C. N. Baldi P. (2022). SSpro/ACCpro 6: Almost perfect prediction of protein secondary structure and relative solvent accessibility using profiles, deep learning and structural similarity. Bioinformatics 38 (7), 2064–2065. 10.1093/bioinformatics/btac019 35108364

[B46] Wallner B. Mirabello C. (2017). Interpred: A pipeline to identify and model protein-protein interactions. Proteins. 85 (6), 1159–1170. 10.1002/prot.25280 28263438

[B47] Wang L. Wang H.-F. Liu S.-R. Yan X. Song K.-J. (2019). Predicting protein-protein interactions from matrix-based protein sequence using convolution neural network and feature-selective rotation forest. Sci. Rep. 9 (1), 9848–9912. 10.1038/s41598-019-46369-4 31285519 PMC6614364

[B48] Yan Y. Huang S.-Y. (2019). Pushing the accuracy limit of shape complementarity for protein-protein docking. BMC Bioinforma. 20 (25), 696. 10.1186/s12859-019-3270-y PMC6929408 31874620

[B49] Zhang Y. Skolnick J. (2005). TM-Align: A protein structure alignment algorithm based on the TM-score. Nucleic acids Res. 33 (7), 2302–2309. 10.1093/nar/gki524 15849316 PMC1084323

