# Supplementary material for: Improving peptide-protein docking with AlphaFold-Multimer using forced sampling
Source: Front Bioinform. 2022 Sep 26;2:959160. doi: 10.3389/fbinf.2022.959160 (PMC9580857; doi:10.3389/fbinf.2022.959160)
Supplement: Supplementary file 1 [file Presentation1.pdf]

Supplementary Information
for
Improving Peptide-Protein Docking with AlphaFold-Multimer using Forced Sampling

Isak Johansson Åkhe Björn Wallner

August 16, 2022

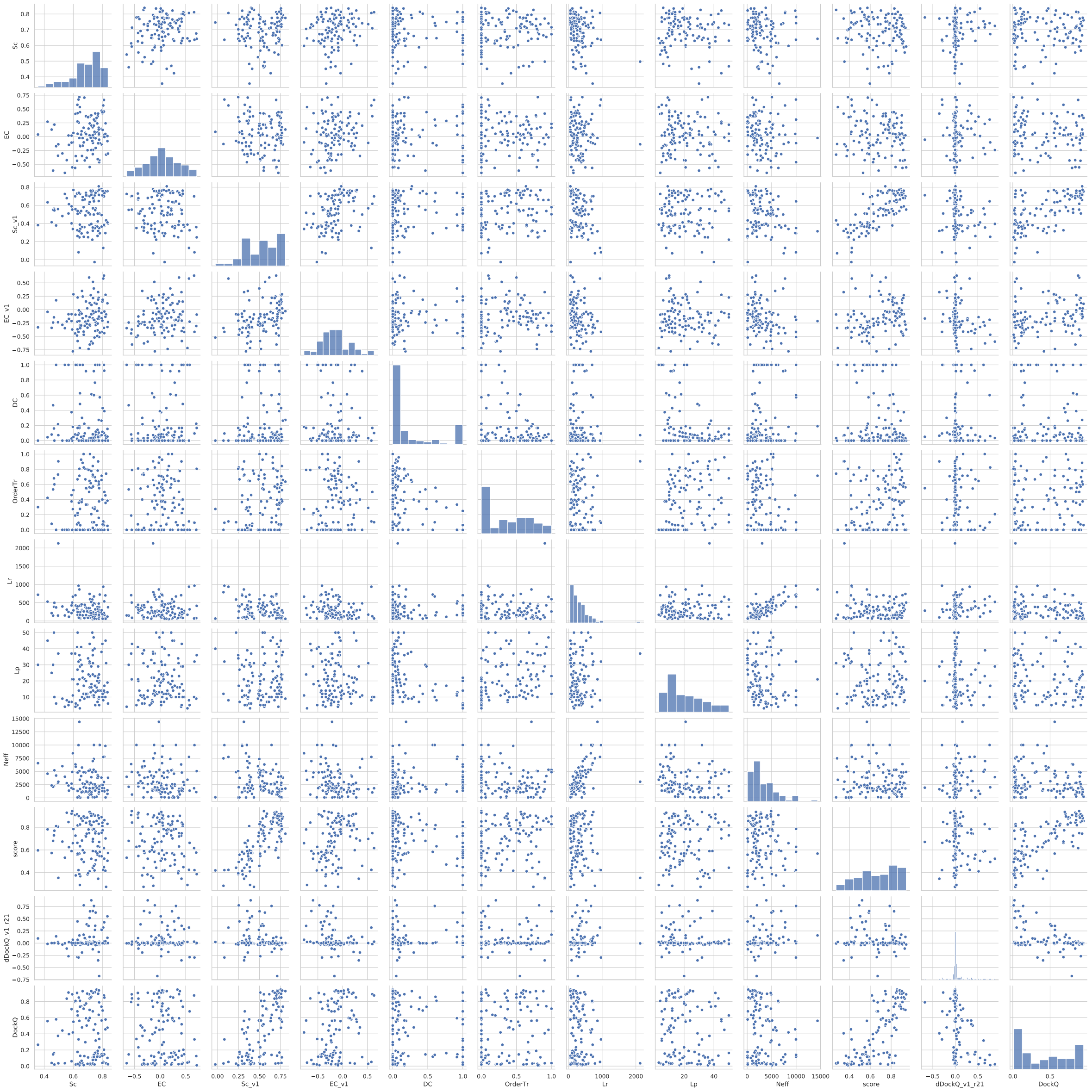

Figure S1: Comparisons of factors that could influence AlphaFold performance with histograms of the features on the diagonal and scatter plots of all features vs. each other off-diagonal. Descriptions of the factors can be found in Table S1. The factors were calculated for the natives and first ranked models by AlphaFold-Multimer-v1 for the 112 non-redundant targets in the dataset.

Factor	Description
Sc	Native surface complementarity
EC	Native electrostatic complementarity
Sc_v1	Model surface complementarity AlphaFold-Multimer v1
EC_v1	Model electrostatic complementarity AlphaFold-Multimer v1
DC	Disorder content
OrderTr	Disorder to order transitions
Lr	Receptor length
Lp	Peptide length
Neff	Effective number of sequence in MSA
score	ranking_confidence score from AlphaFold
dDockQ_v1_r21	DockQ difference between dropout, 21 recycles and standard
DockQ	DockQ score

Table S1: Description of factors

	Sc	EC	Sc_v1	EC_v1	DC	OrderTr	Lr	Lp	Neff	score	dDockQ_v1_r21	DockQ
Sc	1.00	0.17	0.06	0.18	-0.02	-0.00	-0.26	-0.15	-0.11	-0.07	0.13	0.02
EC	0.17	1.00	-0.12	0.21	0.03	0.02	-0.05	-0.05	-0.12	-0.13	-0.02	-0.05
Sc_v1	0.06	-0.12	1.00	0.36	-0.03	0.19	-0.37	0.01	-0.34	0.78	-0.17	0.63
EC_v1	0.18	0.21	0.36	1.00	-0.06	0.06	-0.20	-0.06	-0.24	0.21	-0.27	0.28
DC	-0.02	0.03	-0.03	-0.06	1.00	-0.42	-0.02	-0.40	0.17	-0.15	0.02	-0.06
OrderTr	-0.00	0.02	0.19	0.06	-0.42	1.00	0.02	0.34	-0.19	0.20	0.02	0.17
Lr	-0.26	-0.05	-0.37	-0.20	-0.02	0.02	1.00	0.10	0.60	-0.17	0.12	-0.16
Lp	-0.15	-0.05	0.01	-0.06	-0.40	0.34	0.10	1.00	-0.15	0.03	-0.03	-0.09
Neff	-0.11	-0.12	-0.34	-0.24	0.17	-0.19	0.60	-0.15	1.00	-0.16	0.19	-0.16
score	-0.07	-0.13	0.78	0.21	-0.15	0.20	-0.17	0.03	-0.16	1.00	-0.10	0.74
dDockQ_v1_r21	0.13	-0.02	-0.17	-0.27	0.02	0.02	0.12	-0.03	0.19	-0.10	1.00	-0.31
DockQ	0.02	-0.05	0.63	0.28	-0.06	0.17	-0.16	-0.09	-0.16	0.74	-0.31	1.00

Table S2: Correlations between the factors in Table S1.

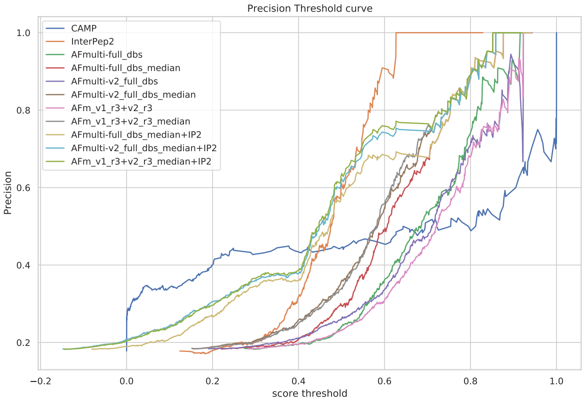

(a) Score threshold versus Precision

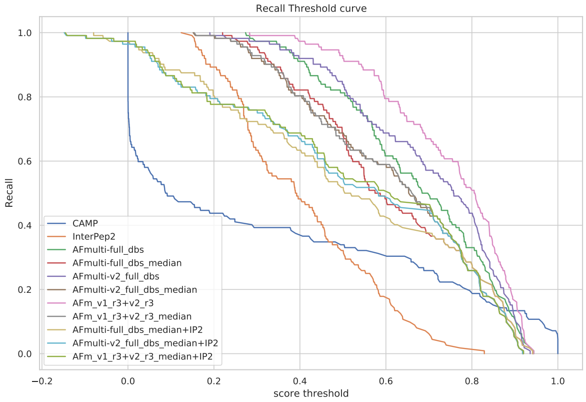

(b) Score threshold versus Recall

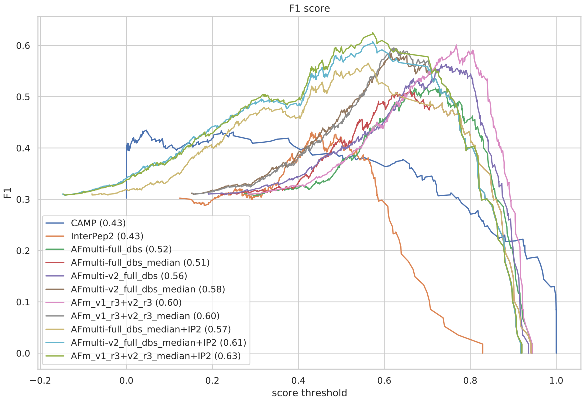

(c) Score threshold versus F1 score

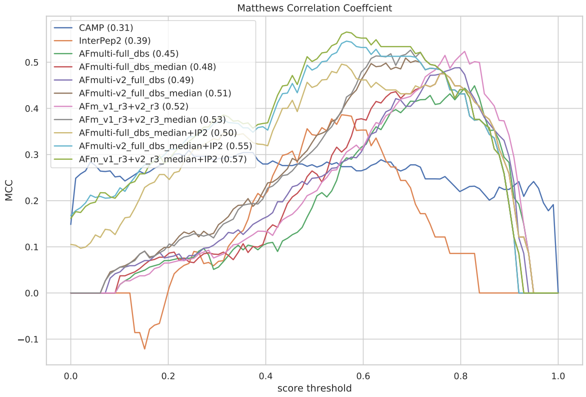

(d) Score threshold versus Matthew's Correlation Coefficient

Figure S2: Plots showing score thresholds versus different metrics.
